# Tebuconazole Remodels 5-*n*-Alk(en)ylresorcinol Profiles in Barley Coleoptiles: Antifungal Properties of the Major Synthetic Homologues

**DOI:** 10.3390/molecules31193398

**Published:** 2026-09-24

**Authors:** Elżbieta G. Magnucka

**Affiliations:** Wrocław University of Environmental and Life Sciences, Department of Plant Protection, Laboratory of Biogeochemistry and Environmental Microbiology, Grunwaldzka St. 53, 50-357 Wrocław, Poland; elzbieta.magnucka@upwr.edu.pl

**Keywords:** 5-*n*-alk(en)ylresorcinols, *Hordeum vulgare*, homologue profile, *Rhizoctonia cerealis*, tebuconazole

## Abstract

5-*n*-Alk(en)ylresorcinols represent a major group of constitutive defense phenolic lipids in cereals; however, the non-target effects of fungicides on their accumulation, homologue profiles, and biological activity remain largely unexplored. This study investigated the effects of tebuconazole, a triazole fungicide widely used for cereal seed treatment, on biomass production, 5-*n*-alk(en)ylresorcinol accumulation, and homologue composition in five barley cultivars, and evaluated the antifungal activity of the predominant synthetic homologues against *Rhizoctonia cerealis*. Two-way ANOVA revealed that tebuconazole treatment had no effect on dry biomass but significantly influenced fresh biomass and total 5-*n*-alk(en)ylresorcinol content in a strongly cultivar-dependent manner. Furthermore, exposure to tebuconazole altered the relative distribution of individual homologues while leaving the qualitative composition largely unchanged, except for minor shifts in the occurrence of trace homologues. Among the individual homologues, C23:0 exhibited the strongest inhibition of *Rhizoctonia cerealis* growth, while C21:0 most effectively suppressed colony regrowth after transfer to compound-free medium. The mixture of the three predominant synthetic homologues exhibited greater antifungal activity than the individual compounds. These findings highlight the importance of considering individual homologue profiles, rather than the total content of the compound alone, when relating the chemical composition of cereal 5-*n*-alk(en)ylresorcinols to their biological activity.

## 1. Introduction

5-*n*-Alk(en)ylresorcinols (ARs), also referred to as resorcinolic lipids, are a group of amphiphilic phenolic compounds widely distributed in plants. Structurally, they consist of a resorcinol ring substituted at position C-5 with a long aliphatic side chain that varies in length and degree of unsaturation [1,2]. Among the higher plants, ARs occur predominantly in members of the *Poaceae* family, where they constitute characteristic compounds of cereal grains. The highest concentrations of ARs have been reported in rye and wheat grains, while barley generally contains lower amounts and exhibits a distinct homologue composition [3,4,5,6,7,8]. On the contrary, considerably less attention has been paid to their accumulation in developing seedling tissues. Although the content and homologue profile of ARs have been investigated in the coleoptiles of rye and wheat [9,10], to date no similar studies have been reported in barley coleoptiles.

Physical and chemical factors influence the accumulation of ARs. Previous studies have demonstrated that pesticides may affect not only the total concentration of these compounds but also the relative abundance of individual homologues [9,11]. The chemicals are of particular interest because they remain in direct contact with the germinating kernels and the developing seedlings during the early stages of plant growth.

Tebuconazole, a systemic triazole fungicide, is widely used as a seed dressing agent in cereal cultivation to protect seedlings against seed- and soil-borne pathogens. Its mode of action is based on inhibition of ergosterol biosynthesis in fungi, resulting in effective suppression of numerous diseases that affect emerging cereal crops [12,13]. Despite the widespread commercial use of tebuconazole in seed protection, its effects on major crops such as barley, and particularly on the accumulation of defensive 5-*n*-alk(en)ylresorcinols, remain entirely unknown.

Resorcinolic lipids are considered biologically active constituents involved in defense against microbial pathogens. Their antibacterial and antifungal properties have been documented for several plant-associated microorganisms, suggesting a potential role in the chemical protection of germinating seeds and young seedlings [14,15,16]. However, the biological significance of the predominant 5-*n*-alk(en)ylresorcinol homologues in barley coleoptiles has not been thoroughly investigated.

Therefore, the objectives of the present study were: (1) to evaluate the effect of tebuconazole seed treatment on the fresh and dry weight (DW) of 5-day-old coleoptiles of several spring barley cultivars, (2) to determine the total concentration and homologue distribution of 5-*n*-alk(en)ylresorcinols in these tissues, and (3) to assess the antifungal activity of selected AR homologues against *Rhizoctonia cerealis*, an important soil-borne pathogen of cereal seedlings.

## 2. Results

### 2.1. Impact of Tebuconazole on the Biomass of Barley Coleoptiles

The fresh biomass of 5-day-old barley coleoptiles differed significantly between the cultivars examined (Table 1).

Under control conditions, Scarlett recorded the highest fresh biomass (17.91 g), while Rataj recorded the lowest (7.14 g).

The effect of tebuconazole on fresh weight varied significantly between cultivars. Fresh biomass increased significantly only in Rataj and Rudzik cultivars, rising from 7.14 to 8.91 g and from 12.98 to 17.24 g, respectively. By contrast, no significant effect of tebuconazole treatment on fresh biomass was detected in cultivars Rabel, Rambo, or Scarlett compared to their respective untreated controls. The dry biomass of coleoptiles was also significantly differentiated between the cultivars studied. In the control treatment, the highest dry biomass was recorded in Scarlett (2.18 g), while the lowest value was found in Rataj (0.91 g). However, exposure to tebuconazole did not significantly affect dry biomass in any of the cultivars examined.

The water content of coleoptiles, calculated from the values of fresh and dry biomass, ranged from 86.59% to 89.39%. The highest water content was found in coleoptiles originating from tebuconazole-treated caryopses of the cultivar Rabel (89.39%), while the lowest value was recorded in untreated Rudzik coleoptiles (86.59%). In most cultivars, the water content was higher after tebuconazole seed treatment than in the corresponding controls.

### 2.2. Effect of Tebuconazole Seed Treatment on Content and Homologue Profile of 5-n-Alk(en)ylresorcinols in Barley Coleoptiles

The total content of 5-*n*-alk(en)ylresorcinols in coleoptiles developed from untreated barley grains differed significantly between the cultivars studied, ranging from 35.60 ± 1.01 mg kg^−1^ DW in the Rabel fodder cultivar to 86.12 ± 1.55 mg kg^−1^ DW in the Scarlett brewing cultivar (Table 2).

Seed treatment with tebuconazole significantly increased the total 5-*n*-alk(en)ylresorcinol content in cultivars Rabel, Rambo, and Rudzik. The strongest response was observed in Rabel, where the total content rose from 35.60 to 66.64 mg kg^−1^ DW, representing an increase of 87.19% relative to the untreated control. By contrast, no significant effect of tebuconazole treatment on the total 5-*n*-alk(en)ylresorcinol content was detected in Rataj or Scarlett cultivars. Analysis of the relative abundance of homologues revealed that long-chain homologues (C19:0, C19:1, and C21:0) constituted the predominant fraction of ARs regardless of cultivar or treatment. In coleoptiles developed from untreated kernels, their contribution ranged from 58.47% to 66.23% of the total AR pool, whereas in coleoptiles originating from grains treated with tebuconazole, it ranged from 48.57% to 69.17%. Within this fraction, the homologue C21:0 was clearly dominant, accounting for 30.90–44.90% of the total ARs. The highest concentration of this component was recorded in Rataj after tebuconazole treatment (35.73 mg kg^−1^ DW), representing 44.90% of the total AR pool.

Very-long-chain homologues (C23:0 and C25:0) represented the second most abundant group of compounds. In coleoptiles originating from untreated caryopses, these constituents represented 18.00–37.50% of total ARs, while after tebuconazole seed treatment, their contribution ranged from 17.70% to 45.71%. Within this fraction, homologue C23:0 consistently predominated over homologue C25:0 in all cultivars and treatments examined. The highest proportion of very-long-chain resorcinolic lipids was observed in Rambo after tebuconazole treatment, where they constituted 45.71% of the total AR pool. In particular, in this cultivar, the contribution of very-long-chain homologues approached that of long-chain homologues (48.57%), a pattern not observed in the remaining cultivars.

Short-chain homologues (C15:0 and C17:0) constituted the smallest fraction of total ARs. In coleoptiles developed from untreated grains, their contribution ranged from 3.90% to 21.71%, while in coleoptiles originating from kernels treated with tebuconazole, it ranged from 5.59% to 33.61% of the total AR pool. The highest proportion of this fraction was detected in Rudzik, both under control conditions (21.71%) and after treatment with tebuconazole (33.61%). This increase was mainly associated with the accumulation of homologue C15:0, whose concentration rose from 4.56 to 21.17 mg kg^−1^ DW. In the remaining cultivars, this constituent was absent or detected only at low concentrations.

Analysis of the degree of saturation demonstrated a clear predominance of saturated homologues. The only unsaturated homologue detected, C19:1, which simultaneously belongs to the long-chain fraction, was found exclusively in coleoptiles developed from untreated caryopses of Rabel and Scarlett. Its concentration reached 2.21 and 0.95 mg kg^−1^ DW, which corresponds to 6.21% and 1.10% of the total AR, respectively. Homologue C19:1 was not detected in coleoptiles originating from grains treated with tebuconazole. Consequently, all resorcinolic lipids detected after tebuconazole exposure were represented exclusively by saturated homologues.

Tebuconazole-induced changes in the homologue profile were specific to the cultivar. In Rabel, tebuconazole seed treatment was associated with both an increase in the total AR content and a rise in the proportion of short-chain homologues from 3.90% to 14.60%. In Rambo, the higher total 5-*n*-alk(en)ylresorcinol content was accompanied by a marked increase in the proportion of very-long-chain homologues, which rose from 25.60% to 45.71%. The most pronounced response occurred in Rudzik, where tebuconazole seed treatment was associated with both elevated total 5-*n*-alk(en)ylresorcinol levels and the highest proportion of short-chain homologues among all cultivars examined. Despite these treatment-dependent shifts in homologue distribution, long-chain homologues, predominantly represented by C21:0, remained the major fraction of ARs in all analyzed samples.

### 2.3. Effects of Cultivar and Tebuconazole Treatment on Coleoptile Biomass and Total AR Content

To further evaluate the effects of cultivar and tebuconazole treatment and their interaction, a two-way analysis of variance (ANOVA) was performed for fresh biomass, dry biomass, and total AR content of barley coleoptiles (Table 3).

The cultivar had a highly significant effect on all three parameters (*p* < 0.001). Treatment with tebuconazole significantly affected fresh biomass (*F* = 50.96, *p* < 0.001) and total AR content (*F* = 240.78, *p* < 0.001), whereas its effect on dry biomass was not significant (*F* = 0.010, *p* = 0.922). A significant cultivar × tebuconazole treatment interaction was observed for fresh biomass (*F* = 31.09, *p* < 0.001) and total AR content (*F* = 40.66, *p* < 0.001), indicating that the response of these parameters to tebuconazole treatment varied between cultivars. In contrast, the cultivar × treatment interaction for dry biomass was not statistically significant (*F* = 2.44, *p* = 0.080). Thus, the two-way ANOVA confirmed that the cultivar was a major source of variation in both biomass and AR accumulation, while the effect of tebuconazole treatment depended on the parameter analyzed and was associated with significant cultivar × treatment interactions for fresh biomass and total AR content.

### 2.4. Effect of Individual 5-n-Alkylresorcinol Homologues on the Radial Growth of Rhizoctonia cerealis

The antifungal activity of the three predominant 5-*n*-alkylresorcinol homologues of barley (C19:0, C21:0, and C23:0) against *Rhizoctonia cerealis* was concentration-dependent (Figure 1).

At the lowest concentration tested (100 mg L^−1^), all homologues caused a minor inhibition of *R. cerealis* radial growth, ranging from 2.51% to 3.39%. Increasing the concentration to 200 mg L^−1^ resulted in a slight further reduction in mycelial growth (10.66–15.57%), with no significant differences observed between the tested compounds at either concentration. Increasing the concentration to 300 mg L^−1^ improved the inhibitory effect of all compounds; however, C23:0 exhibited significantly higher antifungal activity (37.70%) than both C19:0 (17.21%) and C21:0 (20.49%).

The strongest inhibition of fungal growth was observed at 400 mg L^−1^. At this concentration, C23:0 reduced colony diameter by 55.74%, which was significantly greater than inhibition caused by C19:0 (31.97%) and C21:0 (30.33%).

No significant differences were detected between the latter two homologues. Consequently, colony diameter decreased significantly with increasing concentration of each compound, with the greatest reduction consistently observed for C23:0. These results indicate that the antifungal activity of the tested 5-*n*-alkylresorcinol homologues is influenced by both compound concentration and alkyl chain length.

### 2.5. Persistence of the Inhibitory Effect Following Removal of 5-n-Alkylresorcinols

Following the transfer of mycelial plugs from PDA supplemented with 400 mg L^−1^ of individual 5-*n*-alkylresorcinol homologues to fresh AR-free PDA, colony growth varied among treatments (Table 4).

Colonies from cultures exposed to C21:0 had the smallest diameter (3.83 cm), significantly smaller than that of the untreated control (4.13 cm). On the contrary, the colony diameters of cultures previously treated with C19:0 (4.30 cm) and C23:0 (3.97 cm) did not differ significantly from the control.

Consequently, C21:0 exerted the most persistent growth suppression on *R. cerealis* colonies after transfer to AR-free media, reflecting a long-lasting fungistatic or fungicidal-like response under the experimental conditions tested.

### 2.6. Antifungal Activity of Mixtures of the Predominant Synthetic 5-n-Alkylresorcinol Homologues

To determine whether the combination of the three synthetic homologues corresponding to the predominant ARs in barley coleoptiles enhances their antifungal activity, two mixtures containing equal concentrations of C19:0, C21:0 and C23:0 against *R. cerealis* were evaluated.

The antifungal activity of the mixtures was strongly concentration-dependent (Table 5).

Mixture I, which contained each homologue at 100 mg L^−1^ (total AR concentration 300 mg L^−1^), inhibited radial growth of *R. cerealis* by 41.82% (Table 5, Figure 2).Remarkably, this combined activity was higher than that of any individual homologue tested alone at the equivalent total concentration of 300 mg L^−1^ (which yielded 17.21% for C19:0, 20.49% for C21:0, and 37.70% for C23:0; Figure 1). Furthermore, while individual homologues exhibited only marginal growth inhibition (2.51–3.39%) at 100 mg L^−1^ when applied alone, their combined presence at this concentration produced substantial antifungal activity. In contrast, Mixture II, in which each homologue was applied at 300 mg L^−1^ (total concentration 900 mg L^−1^), completely suppressed fungal radial growth (100% inhibition) during exposure; consequently, no fungal colony developed and no representative photograph could be obtained. Because mycelial plugs from this specific mixture treatment were not subjected to a subsequent recovery assay on compound-free medium, this effect is characterized as complete growth suppression during continuous exposure without differentiating between persistent fungistasis and lethal fungicidal activity.

## 3. Discussion

Tebuconazole is a triazole fungicide widely used for cereal protection because of its ability to inhibit ergosterol biosynthesis in fungal pathogens. In addition to their fungicidal activity, triazoles can also modify plant metabolism and stress-related responses, although the magnitude of these effects depends strongly on plant species, genotype, developmental stage, and treatment conditions [17,18]. Consequently, fungicide-induced responses may extend beyond disease control and involve changes in the accumulation of defense-related secondary metabolites. Against this background, the present study examined whether tebuconazole affects the accumulation and biological significance of 5-*n*-alk(en)ylresorcinols during the early stages of barley development. It should be noted that the experimental system utilized in this study involves continuous exposure of germinating caryopses and developing coleoptiles to tebuconazole in a controlled medium. While commercial seed dressings apply a localized dosage onto the caryopsis coat, this laboratory exposure model provides a standardized bioassay to evaluate maximal metabolic response during early seedling growth. Under field conditions, a comparable microenvironment naturally occurs immediately around the germinating seed (the spermatosphere), where the applied fungicide dissolves into the surrounding soil moisture. Treatment of the seeds with tebuconazole exerted only a limited influence on early seedling growth but induced pronounced cultivar-dependent changes in 5-*n*-alk(en)ylresorcinol metabolism. The cultivar-dependent increase in fresh biomass observed in Rataj and Rudzik, in contrast to the lack of significant changes in dry biomass across all cultivars, indicates that this response was primarily driven by increased tissue hydration rather than an increase in organic structural material. Triazole derivatives, including tebuconazole, are known to exert growth-regulating effects by modulating phytohormone crosstalk (such as abscisic acid and cytokinins) and osmoprotectant accumulation, which can alter cell wall elasticity, transpiration rates, and water retention capacity in emerging seedlings [17,18]. Consequently, changes in fresh weight under tebuconazole exposure should be interpreted cautiously as a reflection of the modified water balance in tissue rather than as a true stimulation of seedling growth. Overall, these observations indicate that specialized metabolism responded more sensitively to tebuconazole than growth-related traits. Similar responses have been reported for rye seedlings exposed to chemically unrelated xenobiotics, in which changes in 5-*n*-alk(en)ylresorcinol metabolism preceded measurable alterations in plant growth [11].

Tebuconazole also altered the quantitative distribution of individual homologues, although the response differed markedly between cultivars and no common pattern could be identified. Depending on the genotype, the individual homologues increased, decreased, or remained unchanged, while the qualitative composition of the AR fraction was not affected. Regardless of treatment, C19:0, C21:0 and C23:0 remained the predominant homologues, while minor compounds occurred only sporadically.

The absence of a uniform homologue-specific response aligns with the current understanding of AR biosynthesis, where type III polyketide synthases utilize long-chain acyl-CoA molecules as starter substrates and malonyl-CoA as the elongation unit [19,20,21]. Consequently, the final homologue profile is governed both by alkylresorcinol synthase activity and the pool of available fatty acyl-CoA precursors. However, because plant lipid profiles, acyl-CoA pools, and biosynthetic gene expressions were not measured directly in the present study, any inferred influence on precursor availability remains speculative. Nevertheless, based on established biosynthetic pathways, it can be hypothesized as a working model that tebuconazole may indirectly affect AR homologue distribution by modulating upstream lipid metabolism. Direct quantification of the fatty acid precursor pools, together with transcriptomic profiling, will be essential in future studies to test this hypothesis.

This proposed mechanism is consistent with previous observations in rye seedlings exposed to chemically unrelated xenobiotics. Magnucka et al. [9,11] demonstrated that various pesticides modified both the total 5-*n*-alk(en)ylresorcinol content and the distribution of individual homologues, although the magnitude and direction of these responses depended strongly on experimental conditions. In particular, cycloate was hypothesized to affect AR profiles indirectly by modifying the availability of long-chain acyl-CoA precursors required for AR biosynthesis [9]. Although tebuconazole and cycloate differ fundamentally in their modes of action, both xenobiotics may similarly impact AR profiles through indirect downstream effects on lipid precursor pools. Previous studies characterized the qualitative and quantitative composition of 5-*n*-alk(en)ylresorcinols in mature barley grain [5,6]. The present work extends these observations to developing barley seedlings, demonstrating that although C19:0, C21:0 and C23:0 remain the predominant homologues during early growth, their relative abundance is considerably more responsive to fungicide treatment than in mature tissues. Collectively, these findings indicate that tebuconazole remodels the relative abundance of naturally occurring homologues without changing their qualitative composition, highlighting homologue distribution as a sensitive indicator of metabolic responses to xenobiotic exposure.

The biological relevance of the predominant homologues was further demonstrated by their antifungal activity against *R. cerealis*. Although all three saturated synthetic homologues inhibited fungal growth in a concentration-dependent manner, they differed in their biological effects. C23:0 produced the strongest inhibition of radial mycelial growth during exposure to the compounds, while C21:0 retained the most persistent suppression of colony regrowth after transfer to compound-free medium. These findings indicate that individual homologues are not functionally equivalent. Furthermore, they suggest that the mechanisms responsible for immediate growth inhibition during exposure differ from those governing the persistence of the inhibitory effect upon compound removal, pointing to a more sustained fungicidal-like response for C21:0 under these laboratory conditions. The superior inhibitory activity of C23:0 is consistent with previous reports showing that the antifungal activity of saturated 5-*n*-alkylresorcinols generally increases with the length of the alkyl chain. Patzke et al. [22] observed a similar relationship for several phytopathogenic fungi and proposed that longer-chain homologues interact more effectively with fungal membranes due to their greater hydrophobicity. Although different fungal species and experimental conditions were examined, the present results obtained for *R. cerealis* follow the same general trend.

Previous studies have demonstrated that cereal 5-*n*-alk(en)ylresorcinols contribute to resistance against economically important phytopathogenic fungi [15,23,24]. Altered alkylresorcinol profiles were associated with enhanced resistance of rye seedlings to *R. cerealis* [11], whereas individual saturated homologues inhibited the growth of the closely related *Rhizoctonia solani* [16]. More recently, metabolomic analyzes coupled with biochemometric modeling identified C19:0, C21:0 and C23:0 as the homologues most strongly associated with antifungal activity in cereal extracts [25]. However, because these studies were based on natural extracts or complex metabolite mixtures, they could not distinguish the contribution of individual homologues. Using purified synthetic compounds corresponding to the predominant homologues that occur in barley coleoptiles, the present study provides direct experimental evidence supporting their contribution to antifungal activity.

An additional finding of particular interest was the enhanced antifungal activity of homologue mixtures. When evaluated at an equivalent total active concentration of 300 mg L^−1^, mixture I (100 mg L^−1^ of each homologue) produced greater radial growth inhibition (41.82%) than even the most active single homologue tested alone at 300 mg L^−1^ (C23:0 at 37.70%). Furthermore, while individual homologues exhibited only minimal inhibitory effects at 100 mg L^−1^ when tested alone, their combination at these same low doses yielded pronounced antifungal activity. At a three-fold higher concentration (900 mg L^−1^ total ARs), the mixture achieved complete suppression of fungal growth. However, because mycelial recovery assays were performed only for individual homologues at 400 mg L^−1^, it remains unclear whether the complete inhibition observed for mixture II at a total concentration of 900 mg L^−1^ reflects strong fungistatic activity or an irreversible fungicidal effect. Overall, these results indicate that the antifungal efficacy of naturally co-occurring homologues involves joint additive or synergistic effects that cannot be predicted solely from individual compound assays, highlighting the functional importance of the natural 5-*n*-alkylresorcinol profile as a whole.

Collectively, these findings demonstrate that both the structural identity of individual homologues and their joint presence govern intrinsic antifungal activity under in vitro conditions. However, because the present study evaluated purified synthetic compounds in medium assays without direct seedling infection trials, these in vitro observations cannot be directly extrapolated to complex in planta defense mechanisms. Although tebuconazole remodels the relative abundance of predominant homologues in barley coleoptiles, confirmation of whether such metabolic shifts functionally alter host resistance against *Rhizoctonia cerealis* will require dedicated in planta pathogen challenge assays in future studies. Consequently, evaluating individual homologue profiles alongside the total AR content provides a more refined framework for formulating hypotheses on the defensive role of these phenolic lipids. 

## 4. Materials and Methods

### 4.1. Chemicals and Materials

Orius Extra 02WS (a commercial formulation containing 2% tebuconazole; IUPAC name: (*RS*)-1-(4-chlorophenyl)-4,4-dimethyl-3-(1*H*-1,2,4-triazol-1-ylmethyl)pentan-3-ol) was purchased from Nufarm Poland (Warsaw, Poland). All solvents were supplied by Avantor Performance Materials Poland S.A. (Gliwice, Poland). Synthetic 5-*n*-alkylresorcinol homologues (C19:0, C21:0, and C23:0), Fast Blue B salt (DBB), potato dextrose agar (PDA), Tween 80, sodium hypochlorite, and preparative thin-layer chromatography (TLC) plates were obtained from Merck KGaA (Darmstadt, Germany).

In this study, certified seed lots of five spring barley cultivars (*Hordeum vulgare* L.), namely Scarlett, Rabel, Rambo, Rataj, and Rudzik, were used. Voucher information for all cultivars is available from the Research Centre for Cultivar Testing (COBORU, Słupia Wielka, Poland).

*R. cerealis* strain IOR1794 was obtained from the Pathogen Collection of the Institute of Plant Protection—National Research Institute (Poznań, Poland).

### 4.2. Plant Experiment

Barley kernels (25 g per replicate, representing approximately 500–600 caryopses) were surface-disinfected by immersion in 0.1% (*v*/*v*) Tween 80 for 20 min, followed by incubation in 5% (*v*/*v*) sodium hypochlorite for 50 min. The kernels were dressed with a tebuconazole solution (10 mg L^−1^) and then placed in growth chambers on sterile tissue paper moistened with the same solution. The control kernels received only sterile distilled water. The barley seedlings were grown at 22 ± 2 °C under continuous illumination (200 µmol m^−2^ s^−1^ PPFD; Fluora fluorescent lamps, Osram, Germany). Germination rates ranged from 70 to 80% across the tested cultivars and treatments. For each cultivar and treatment, seedlings were grown in three chambers per experimental run on three independent temporal runs. For fresh and dry biomass determinations, 5-day-old coleoptiles harvested from each chamber were weighed gravimetrically.

### 4.3. Isolation, Purification, and Determination of 5-n-Alk(en)ylresorcinol Level

Resorcinolic lipids were extracted and purified from coleoptiles previously dried at 50 ± 5 °C, according to the method described by Magnucka et al. [9]. For each of the three independent biological experiments (n=3), the extracted solutions obtained from the three chambers of a given cultivar and treatment were pooled after extraction to yield a single representative biological extract. The total 5-*n*-alk(en)ylresorcinol content was determined using the colorimetric assay of Gajda et al. [26]. Quantification was based on a calibration curve constructed with 5-*n*-heneicosylresorcinol (C21:0) as an external standard in the range of 1–10 µg (R^2^ = 0.975). All colorimetric assays were performed in technical triplicates for each pooled biological extract (n=3). 

### 4.4. Homologue Composition Analysis

The homologue profile of resorcinolic lipids was determined by gas chromatography coupled with electron ionization mass spectrometry (GC/EI-MS) according to a previously described method [9]. GC/EI-MS analyses were performed on each of the three independent biological extracts (n=3). Identification of individual homologues was based on the presence of the molecular ion (M^+^) and the characteristic fragment ion at *m/z* 268, which is diagnostic for 5-*n*-alk(en)ylresorcinols. Saturated homologues were identified by their retention times and molecular ions as follows: C15:0, 9.3 min (*m/z* 464, M^+^); C17:0, 10.4 min (*m/z* 492, M^+^); C19:1, C19:0, 11.6 min (*m/z* 520, M^+^); C21:0, 12.7 min (*m/z* 548, M^+^); C23:0, 13.8 min (*m/z* 576, M^+^); and C25:0, 14.9 min (*m/z* 604, M^+^). The unsaturated homologue C19:1 was identified by its molecular ion at *m/z* 518 (M^+^) and a retention time distinct from that of the corresponding saturated homologue C19:0.

### 4.5. Antifungal Activity of the Predominant 5-n-Alkylresorcinol Homologues

Tebuconazole altered the homologue composition of resorcinolic lipids in barley coleoptiles. Regardless of cultivar and treatment, long-chain homologues predominated in the AR pool. Therefore, representative long-chain homologues (C19:0 and C21:0) together with the predominant very-long-chain homologue (C23:0) were selected for antifungal assays against *R. cerealis* strain IOR1794, a major soil-borne pathogen of barley seedlings.

The solutions of individual homologues were prepared in acetone and applied to the surface of solidified potato dextrose agar (PDA) to obtain final concentrations of 100, 200, 300, and 400 mg L^−1^. The same fixed volume of acetone was used for all treatments and for the solvent control (CS). The control plates received only acetone. After solvent evaporation (1.5 h in a laminar flow cabinet), a 3 mm agar plug excised from the margin of an actively growing colony of *R. cerealis* was placed in the center of each plate. The plates were incubated at 22 °C in the dark. Fungal growth was monitored at 12 h intervals until the mycelium in the solvent control covered approximately 95–98% of the surface area. Percent inhibition of mycelial growth was calculated relative to the solvent-treated control. To distinguish between the fungistatic and fungicidal effects of the homologues tested, a mycelial recovery assay was performed. Agar plugs were removed from actively growing margins of colonies exposed to the highest tested concentration (400 mg L^−1^) of each homologue and transferred to fresh PDA plates without 5-*n*-alkylresorcinols. The plates were incubated under the same conditions as described above, and colony regrowth was assessed after 72 h of incubation, corresponding to the time when control reached approximately 95–98% coverage of the Petri dish. The persistence of growth inhibition after transfer to compound-free PDA was interpreted as reflecting the relative fungistatic or fungicidal nature of the tested compounds. Recovery of fungal growth indicated a predominantly fungistatic effect, whereas maintenance of growth inhibition suggested a more persistent, fungicidal-like action under the experimental conditions. Furthermore, the antifungal activity of two mixtures containing all three homologues (C19:0, C21:0, and C23:0) was evaluated using the same solvent application procedure. In Mixture I, each homologue was present at 100 mg L^−1^, resulting in a total AR concentration of 300 mg L^−1^, while in Mixture II, each homologue was present at 300 mg L^−1^, resulting in a total AR concentration of 900 mg L^−1^.The experiment was carried out independently three times, with three Petri plates per treatment in each experiment.

### 4.6. Statistical Analyses

Statistical analyses were performed using STATISTICA software (version 13; TIBCO Software Inc., Palo Alto, CA, USA). Differences between cultivar means within each treatment were evaluated using a one-way analysis of variance (ANOVA), followed by Duncan’s multiple range test at a significance level of *p* ≤ 0.05. In addition, a two-way ANOVA was performed to assess the main effects of cultivar and tebuconazole treatment, as well as their interaction (cultivar × treatment), on fresh biomass, dry biomass and the total 5-*n*-alk(en)ylresorcinol content of barley coleoptiles. The results of the two-way ANOVA are presented as *F*-values and corresponding *p*-values in Table 3. Differences were considered statistically significant at *p* < 0.05.

## 5. Conclusions

Tebuconazole remodelled the 5-*n*-alk(en)ylresorcinol profile of the developing barley seedlings in a cultivar-dependent manner by altering the relative abundance of the predominant homologues while leaving the qualitative composition largely unchanged except for minor trace homologue shifts. The predominant synthetic homologues differed markedly in their antifungal activity against *R. cerealis*—exhibiting distinct levels of immediate growth inhibition and persistent fungicidal-like suppression—while their combination exhibited greater antifungal activity than the individual compounds. These findings demonstrate that the in vitro antifungal efficacy of cereal ARs depends not only on their total concentration but also on the specific distribution of individual homologues. While direct seedling infection assays are required to confirm these effects in planta, this work highlights the necessity of homologue-specific profiling when evaluating the bioactivity of secondary metabolites in plant–xenobiotic interactions.

## Figures and Tables

**Figure 1:**
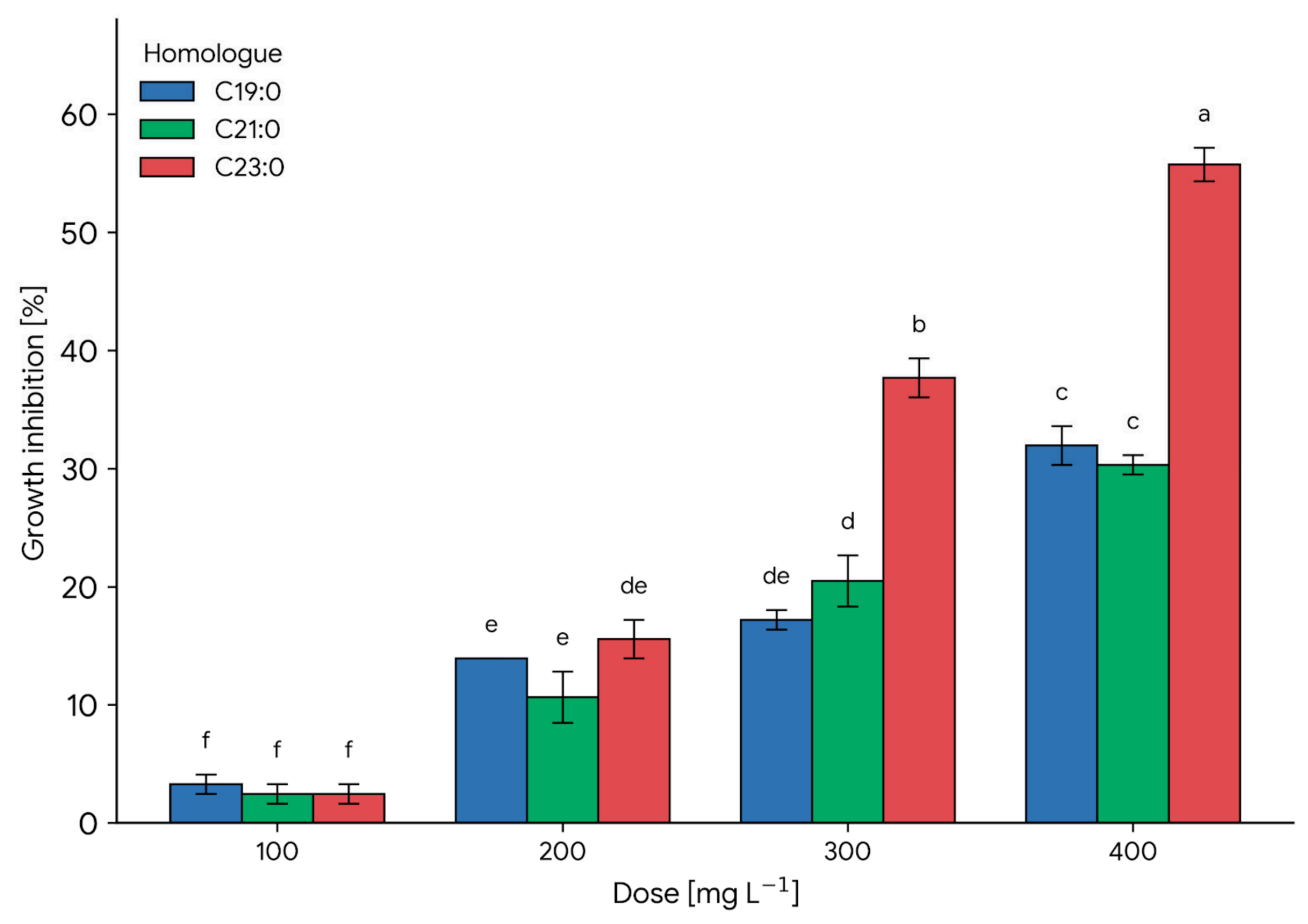
Antifungal activity of the predominant 5-*n*-alkylresorcinol homologues (C19:0, C21:0, and C23:0) against *Rhizoctonia cerealis* at doses ranging from 100 to 400 ${\mathrm{mg}\;L}^{-1}$. Data represent mean values ± SE of three independent experiments. Means with different letters (a–f) are significantly different at p≤0.05 according to Duncan’s multiple range test.

**Figure 2:**
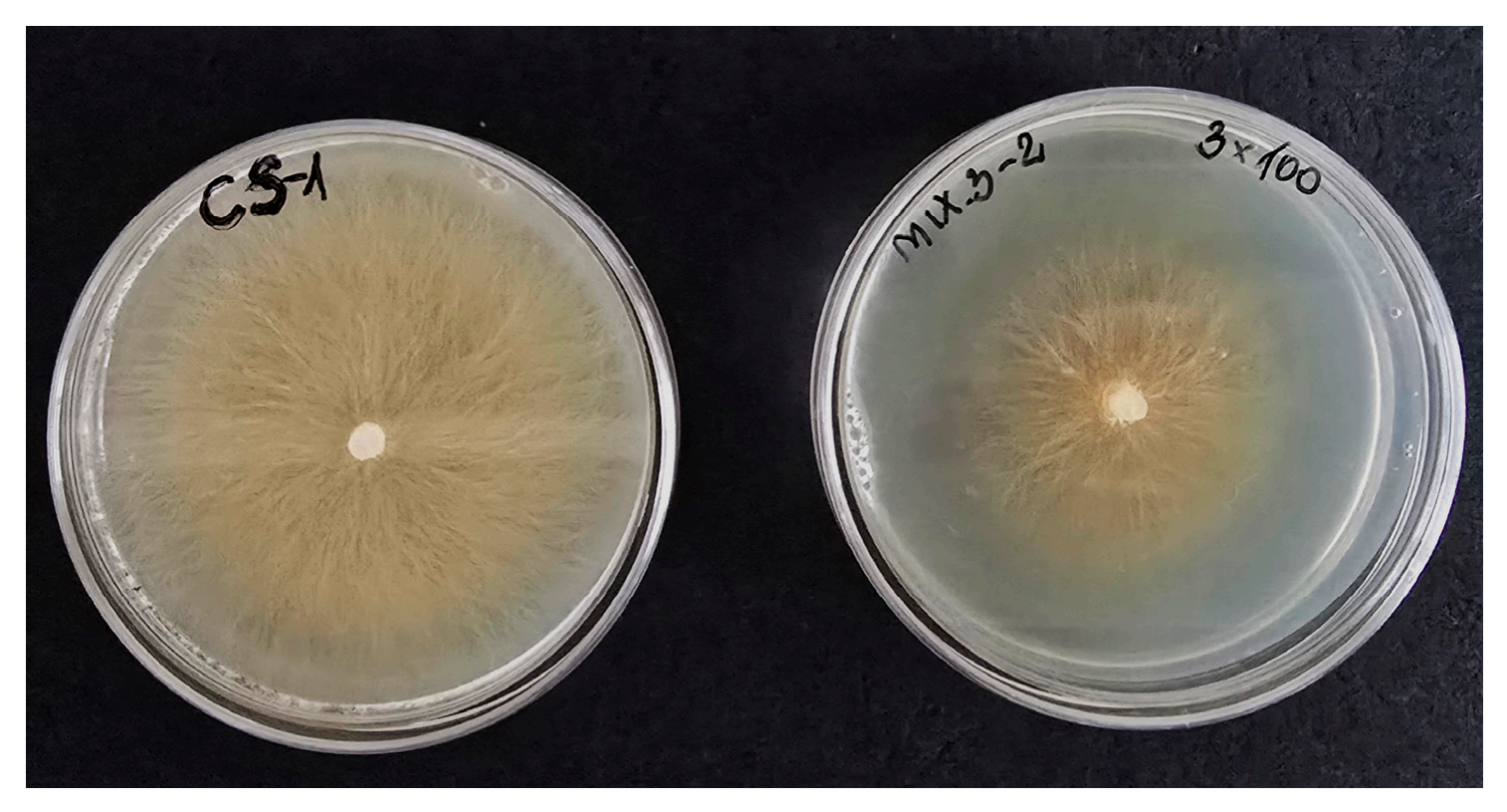
Representative photographs of *Rhizoctonia cerealis* cultures showing the inhibitory effect of Mixture I (100 mg L^−1^ each of synthetic C19:0, C21:0 and C23:0) on radial mycelial growth relative to the solvent control (CS).

**Table 1:** Effect of tebuconazole on the fresh and dry weight of barley coleoptiles.

Cultivar	Treatment	Coleoptile Weight [g]	
		Fresh Biomass	Dry Biomass
Rabel	Control	13.53 ± 0.34 b	1.56 ± 0.18 cd
	Fungicide	13.76 ± 0.29 b	1.46 ± 0.12 ce
Rambo	Control	11.81 ± 0.30 c	1.55 ± 0.13 cd
	Fungicide	11.52 ± 0.27 c	1.23 ± 0.10 def
Rataj	Control	7.14 ± 0.08 e	0.91 ± 0.06 f
	Fungicide	8.91 ± 0.17 d	1.12 ± 0.01 ef
Rudzik	Control	12.98 ± 0.31 b	1.74 ± 0.03 bc
	Fungicide	17.24 ± 0.10 a	1.99 ± 0.04 ab
Scarlett	Control	17.91 ± 0.11 a	2.18 ± 0.14 a
	Fungicide	17.55 ± 0.31 a	2.07 ± 0.16 ab

Mean values ± SE of three independent experiments. Values followed by different letters differ significantly at *p* ≤ 0.05 according to Duncan’s multiple range test; fresh biomass and dry weight were analyzed separately.

**Table 2:** Effect of fungicide on the content and composition of 5-*n*-alk(en)ylresorcinol homologues in barley coleoptiles.

Cultivar	Treatment	AR Content [mg kg^−1^ DW]	Individual Homologue Content [mg kg^−1^ DW]						
			C15:0	C17:0	C19:1	C19:0	C21:0	C23:0	C25:0
Rabel	Control	35.60 ± 1.01 e	0.00	1.39	2.21	7.65	11.00	7.05	6.30
	Tebuconazole	66.64 ± 1.80 c	0.00	9.73	0.00	16.33	17.19	10.80	12.59
Rambo	Control	63.82 ± 1.81 c	0.00	5.23	0.00	21.12	21.13	11.42	4.92
	Tebuconazole	82.72 ± 1.04 ab	0.00	4.72	0.00	12.56	27.63	24.57	13.24
Rataj	Control	79.23 ± 0.51 b	0.00	13.55	0.00	24.64	26.78	10.85	3.41
	Tebuconazole	79.57 ± 1.49 b	0.00	4.45	0.00	19.34	35.73	15.12	4.93
Rudzik	Control	57.61 ± 1.34 d	4.56	7.95	0.00	15.61	18.09	5.93	5.47
	Tebuconazole	78.70 ± 1.82 b	21.17	5.28	0.00	18.49	19.83	9.37	4.56
Scarlett	Control	86.12 ± 1.55 a	0.00	6.72	0.95	22.56	30.23	13.18	12.48
	Tebuconazole	87.37 ± 1.80 a	4.37	5.15	0.00	18.52	27.00	22.37	9.96

Data are presented as mean values calculated from three independent biological replicates. The SE values for individual homologues were below 5% of the corresponding mean values. Values followed by different letters are significantly different at *p* ≤ 0.05 according to Duncan’s multiple range test. DW, dry weight.

**Table 3:** Two-way ANOVA of the effects of cultivar and tebuconazole treatment on coleoptile biomass and total AR content.

Parameter	Factor	*F*-Value	*p*-Value
Fresh biomass	Cultivar	433.31	<0.001
	Tebuconazole treatment	50.96	<0.001
	Cultivar × treatment	31.09	<0.001
Dry biomass	Cultivar	29.33	<0.001
	Tebuconazole treatment	0.01	0.922
	Cultivar × treatment	2.44	0.080
Total AR content	Cultivar	165.32	<0.001
	Tebuconazole treatment	240.78	<0.001
	Cultivar × treatment	40.66	<0.001

Two-way ANOVA was performed with cultivar and tebuconazole treatment (untreated control vs. tebuconazole-treated) as fixed factors. *F*, *F*-statistic; *p*, probability value. Statistical significance was set at *p* < 0.05.

**Table 4:** *Rhizoctonia cerealis* colony regrowth after transfer of mycelial plugs from PDA containing 400 mg L^−1^ of individual 5-*n*-alkylresorcinol homologues to fresh PDA without AR.

Treatment	Colony Diameter [cm]
C19:0	4.30 ± 0.00 a
C21:0	3.83 ± 0.07 c
C23:0	3.97 ± 0.09 bc
Control	4.13 ± 0.09 ab

Mean values ± SE from three independent experiments evaluated after 72 h of incubation on AR-free PDA. Values followed by different letters are significantly different at *p* ≤ 0.05 according to Duncan’s multiple range test.

**Table 5:** Effect of mixtures of the three predominant synthetic 5-*n*-alkylresorcinol homologues (C19:0, C21:0 and C23:0) on inhibition of radial growth of *Rhizoctonia cerealis*.

Treatment	Composition	Total Concentration [mg L^−1^]	Radial Growth Inhibition [%]
Mixture I	C19:0 + C21:0 + C23:0 (100 mg L^−1^ each)	300	41.82 ± 0.03 b
Mixture II	C19:0 + C21:0 + C23:0 (300 mg L^−1^ each)	900	100 ± 0.00 a

Values represent means ± SE of three independent experiments. Different letters indicate significant differences according to Duncan’s multiple range test (*p* ≤ 0.05).

## Data Availability

The original contributions presented in this study are included in the article. Further inquiries can be directed to the corresponding author.
